# Levetiracetam Prophylaxis Therapy for Brain Tumor-Related Epilepsy (BTRE) Is Associated With a Higher Psychiatric Burden

**DOI:** 10.3389/fneur.2021.806839

**Published:** 2022-01-11

**Authors:** Fedele Dono, Stefano Consoli, Giacomo Evangelista, Annalisa Ricci, Mirella Russo, Claudia Carrarini, Angelo Di Iorio, Laura Bonanni, Francesca Anzellotti, Marco Onofrj, Stefano L. Sensi

**Affiliations:** ^1^Department of Neuroscience, Imaging and Clinical Science, “G. d'Annunzio” University of Chieti-Pescara, Chieti, Italy; ^2^Behavioral Neurology and Molecular Neurology Units, Center for Advanced Studies and Technology (CAST), “G. d'Annunzio” University of Chieti-Pescara, Chieti, Italy; ^3^Department of Medicine and Ageing Sciences, “G. d'Annunzio” University of Chieti-Pescara, Chieti, Italy; ^4^Department of Neurology, “SS Annunziata” Hospital, Chieti, Italy; ^5^Institute for Mind Impairments and Neurological Disorders (iMIND), University of California, Irvine, Irvine, CA, United States

**Keywords:** AMPA, glutamate, brain tumor epilepsy, psychiatry, side effect

## Abstract

**Purpose:** Brain tumor-related epilepsy (BTRE) is a condition characterized by the development of seizures in the context of an undergoing oncological background. Levetiracetam (LEV) is a third-generation anti-seizure medication (ASM) widely used in BTRE prophylaxis. The study evaluated LEV neuropsychiatric side effects (SEs) in BTRE prophylaxis.

**Method:** Twenty-eight patients with brain tumors were retrospectively selected and divided into two groups. In one group, we evaluated patients with a BTRE diagnosis using LEV (BTRE-group). The other group included patients with brain tumors who never had epilepsy and used a prophylactic ASM regimen with LEV (PROPHYLAXIS-group). Neuropsychiatric SEs of LEV were monitored using the Neuropsychiatric Inventory Questionnaire (NPI-Q) at the baseline visit and the 6- and 12-month follow-up.

**Results:** Eighteen patients of the BTRE-group and 10 patients of the PROPHYLAXIS-group were included. Compared to the BTRE-group, the PROPHYLAXIS-group showed a higher severity of neuropsychiatric symptoms. According to Linear Mixed Models (LMM), a multiplicative effect was observed for the interaction between group treatment and time. For the caregiver distress score (CDS), only a time-effect was observed.

**Conclusion:** Prophylactic ASM with LEV is associated with an increased frequency of neuropsychiatric SE. Accurate epileptological evaluations in patients with brain tumors are mandatory to select who would benefit most from ASM.

## Introduction

Brain tumor-related epilepsy (BTRE) is one of the most frequent neurological manifestations in the context of brain tumors. Seizures are frequently the onset symptoms in up to 40% of patients (1) with brain tumors. The prevalence of BTRE in patients with supratentorial brain tumors is up to 75%, with the highest percentage in cases of low-grade astrocytoma (2, 3). Treatment of BTRE is challenging due to the efficacy of anti-seizure medications (ASM) and the distinct side-effects occurring in these patients that differ from non-oncological patients (4). Data on new ASM indicate a percentage of side-effects ranging from 7 to 44.4% (4).

According to some studies, the high prevalence of BTRE in patients suffering from brain tumors justifies the prophylactic use of ASM. The rationale for the procedure relates to the higher risk of developing BTRE, especially in association with brain surgery (5). The incidence of seizures is estimated to be 15–20% in patients who underwent non-traumatic, supratentorial craniotomy. According to expert opinion, the use of ASM in patients with supratentorial brain tumors should consider (6) risk-benefits assessment. According to the literature, ASM prophylactic treatment is generally recommended during the perioperative period, starting from brain tumor diagnosis and prolonged from 1 week to more than 12 months after brain surgery (7).

Levetiracetam (LEV) is a third-generation ASM that mainly blocks the SV2A presynaptic protein and decreases levels of excitatory neurotransmitters. Data in the literature highlight that LEV is associated with a favorable outcome in BTRE with a consistent reduction in seizure frequency (4, 8). LEV is also frequently used in BTRE prophylaxis, thanks to easy titration and low interaction with anti-neoplastic treatments (9). Most common side effects (SEs) of LEV include agitation, irritability, and aggressiveness. Tolerability and SEs of LEV in the context of BTRE and prophylactic treatment have been extensively evaluated throughout standard SE scales, such as Adverse Event Profile (AEP) scale and Quality of Life Questionnaire-35 (QOL-35) (4). However, these scales do not assess the psychiatric profiles of patients.

This study evaluated the psychiatric tolerability of LEV when used in prophylactic treatment compared to treatment of patients suffering from BTRE.

## Methods

### Patient Demographics and Clinical Features

Adult patients with primitive brain tumors were retrospectively selected from the database of inpatients referred to the Neurology Clinic of “G. d'Annunzio” University of Chieti-Pescara from September 2018 to June 2020. The selection was made according to the following inclusion criteria: Mini-Mental State Examination (MMSE) score >24 at the time of brain tumor diagnosis, no history of psychiatric disorders, and no treatment with medications that interfere with behavioral functioning. As per routine protocol, all patients with brain tumors had undergone neuropsychiatric evaluations with Neuropsychiatric Inventory Questionnaire (NPI-Q) at the time of brain tumor diagnosis and the 6–12 month follow-up visits. Furthermore, patients had undergone a standard 21-channel-electroencephalogram (EEG) recording to confirm the diagnosis of BTRE. Diagnosis of epilepsy was reviewed based on the clinical and electrophysiological information according to International League Against Epilepsy (ILAE) diagnostic criteria. Independently of BTRE diagnosis, all patients included had been treated with oral administration of LEV. Patients were divided into two groups: patients with BTRE treated with LEV (BTRE-group) and patients without BTRE treated with LEV as prophylactic ASM (PROPHYLAXIS-group). As per standard clinical protocols, BTRE prophylaxis treatment usually lasts 12 months. Demographics, clinical, radiological, and neurophysiological data were compared between groups. In addition, mortality at 12 months was assessed in both groups. The study was approved by the local ethics committee (“G. d'Annunzio” University of Chieti-Pescara, Protocol code 2098. 11/06/2020, Protocol “Neurodem” 26/7/2018, Emend 2/8/2018). The patients/participants provided their written informed consent to participate in the study. If the patient could not read, write, or hear, informed consent was obtained from the legal guardian(s) of the patient. The present study was performed in agreement with the Declaration of Helsinki.

### Neuropsychiatric Evaluation

The NPI-Q is a semi-structured clinician interview of caretakers that rates the severity and frequency of disturbance in 12 symptom domains (delusions, hallucinations, agitation, depression, anxiety, euphoria, apathy, disinhibition, irritability, aberrant motor activity, sleep disturbance, and eating disorders). For each domain, the overall impact is estimated as the product of the frequency of the psychiatric symptom (scores range 0–4) and its severity (scores range 0–3). In addition, caregivers rate the associated impact of the symptom manifestations on them using a five-point scale (caregiver distress scale, CDS).

The Neuropsychiatric Inventory Questionnaire total score represents the sum of all 12 sub-scores and ranges between 0 and 144. Scoring in subscales of the NPI-Q has been shown to strongly correlate with those in other well-validated symptomatic scales, such as the Hamilton Rating Scale for Depression (10). According to the literature, NPI-Q could be crucial in behavioral assessment in patients with brain tumors. Indeed, NPI-Q in brain tumor patients seems more appropriate than behavioral inventories implemented for psychiatric populations (e.g., Mini-International Neuropsychiatric Interview—M.I.N.I.). Furthermore, administering by-proxy neuropsychiatric tests allows detecting behavioral disturbances in patients with language impairment or anosognosia (11).

The NPI-Q was administered to the spouse or to a close first-degree relative who lived with the patient.

### Statistical Analysis

Data are reported as the median plus interquartile range (IQR) or absolute number and percentage for continuous, or categorical and dichotomous variables, respectively. Differences between groups were compared using general linear models for continuous variables and with the χ^2^ test for categorical and dichotomous variables. Differences in the frequency of clinical manifestations between two groups were assessed with the χ^2^ test. Logistic Regression Models were used to produce odds ratio (OR), and 95% CI, to assess ASM prophylactic therapy association with mortality. To evaluate variations in scores related to the NPI-Q scale according to time (baseline, 6 and 12 months follow-up) and group (BTRE-group and PROPHYLAXIS-group), Linear Mixed Models (LMMs) were used. The main effects of fixed factors and their respective interactions were assessed by model comparisons (Likelihood Ratio Tests). The intercept was added as random factors with uncorrelated random intercepts and slopes within participant and time. LMMs highlight interactive effects among predictors (i.e., whether ASM prophylactic therapy and the course of time synergically impact on NPI-Q total score of CDS score) or unravel individual effects of each predictor on a given outcome (i.e., whether ASM prophylactic therapy, independently of time, affects the risk for increased NPI-Q total score or CDS score). Analyses were conducted using SAS 9.4 (SAS Institute Inc., Cary, NC, USA). All statistical tests were two-sided, and statistical significance was defined at *p* < 0.05.

## Results

### Demographics

Eighteen adult patients (mean age: 46.9 ± 15.5; men: 11) with BTRE (BTRE-group) and 10 patients (mean age: 60.7 ± 16; men: 5) under prophylactic treatment with LEV (PROPHYLAXIS-group) were eligible for the study. At the study entry, no patients showed psychiatric symptoms or cognitive impairment as assessed by the NPI-Q and MMSE scores. Sixteen patients in the BTRE-group (16/18, 89%) and 9 patients in the PROPHYLAXIS-group (9/10, 90%) were married and living together with their spouses. Two patients in the BTRE-group (2/18, 11%) and one patient in the PROPHYLAXIS-group (1/10, 10%) lived with their relatives. Two patients in the BTRE-group (2/18, 11%) and five patients in the PROPHYLAXIS-group (5/10, 50%) died due to tumor progression during follow-up. Sixteen patients in the BTRE-group (16/18, 89%) and five patients in the PROPHYLAXIS-group (5/10, 50%) completed the 12-month follow-up. Demographic data are summarized in Table 1.

**Table 1:** Demographics and brain tumor characteristics and treatment.

	**BTRE-group *(n = 18)***	**PROPHYLAXIS-group *(n = 10)***	
Age	46.9 ± 15.5	60.7 ± 16	*p = 0.04*
Sex	11M 7F	5M 5F	*p = 0.67*
Baseline KPS [median (IQR)]	1 (0.9–1)	1 (0.9–1)	*p = 0.84*
6-months KPS [median (IQR)]	1 (0.9–1)	1 (0.9–1)	*p = 0.84*
12-months KPS [median (IQR)]	0.9 (0.8–1)	0.9 (0.8–1)	*p = 0.13*
Tumor hystology	° Glioblastoma (WHO IV): 10 ° Anaplastic astrocitoma (WHO III): 1 ° Low grade astrocitoma (WHO II): 4 ° Oligoastrocitoma: 3	° Glioblastoma (WHO IV): 5 ° Anaplastic astrocitoma (WHO III): 2 ° Low grade astrocitoma (WHO II): 2 ° Cerebral gliosarcoma: 1	*p = 0.67*
Localization	° Frontal: 7 ° Temporal: 7 ° Parietal: 4	° Frontal: 3 ° Temporal: 3 ° Parietal: 4	*p = 0.79*
Dimension	° <5 cm: 9 ° >5 cm: 9	° <5 cm: 7 ° >5 cm: 3	*p = 0.58*
Lateralization	° Right 10 ° Left 8	° Right 4 ° Left 6	*p = 0.62*
Type of surgery	° GTR: 8 ° STR: 10	° GRS: 5 ° STR: 5	*p = 0.67*
12-months tumor relapse	2	5	*p = 0.03*
CT/RT treatment	14	8	*p = 0.27*
12-months tumor mortality	2	5	*p = 0.03*

*KPS, Karnoski Performance status; GRS, gross tumor resection; STR, sub-total tumor resection; CT, chemotherapy; RT, radiotherapy*.

### Seizure Prevalence and Electrophysiological Assessment

In the BTRE-group, focal seizures represented the onset symptom of underlying tumor pathology in 14 patients (78%). Focal-to-bilateral seizures were reported in 4 patients (4/18, 22%). EEG evaluation in BTRE group showed focal slow activity in 9 patients (9/18, 50%), epileptiform discharges in 3 patients (3/18, 17%), lateralized periodic discharges in 1 patient (1/18, 6%), and normal EEG in 4 patients (4/18, 22%). In the PROPHYLAXIS-group, EEG analysis revealed focal slow activity in 3 patients (3/10, 30%), lateralized periodic discharges in 1 patient (1/10, 10%), whereas 4 patients showed a normal EEG (4/10, 40%). The median LEV daily dose in the BTRE-group was 2,000 mg (IQR: 1,500–2,500 mg) and 2,000 mg (IQR: 1,000–2,000 mg) in the PROPHYLAXIS-group. No differences in the titration schedule were observed when comparing the two groups. Dose adjustment was performed according to seizure frequency of the patients during the follow-up in 2 patients in the BTRE group, who experienced seizure recurrence during follow-up. Serum levels of LEV were tested and always resulted within the normal range. Blood tests showed no abnormal findings in both groups. In particular, normal levels of creatinine clearance were observed. At the 12 months follow-up visit, seizure freedom was detected in 16 patients (16/18, 89%), whereas 2 patients (2/18, 11%) presented a reduction >50%. No seizures have been reported in the PROPHYLAXIS-group for the whole length of the follow-up period. Discontinuation of ASM or treatment dose reduction due to the onset of AE was observed neither in the BTRE-group nor in the PROPHYLAXIS-group.

Seizure prevalence and electrophysiological assessment features are summarized in Table 2.

**Table 2:** Seizure prevalence and electrophysiological assessment features.

	**BTRE-group *(n = 18)***	**PROPHYLAXIS-group *(n = 10)***
Seizure type	° Focal: 14 ° Focal-to-bilateral: 4	NA
Seizure onset concomitant to brain tumor diagnosis	4	NA
Electroencephalogram (EEG)	° Focal slow: 9 ° EDs: 3 ° LPDs: 1 ° Normal: 4 ° Not available: 1	° Focal slow: 3 ° LPDs: 1 ° Normal: 4 ° Not available: 2
LEV dose [median (IQR)]	2,000 mg (1,500–2,500)	2,000 mg (1,000–2,000)
12-months seizure freedom (number of patients)	16	NA

*EDs, epileptic discharges; LPDs, lateralized periodic discharges*.

### Brain Tumor Characteristics and Treatment

Histological evaluation revealed a WHO IV glioblastoma in 10 patients in the BTRE-group (10/18, 55%) and 5 patients in the PROPHYLAXIS-group (5/10, 50%). Frontal localization was reported in 7 patients in the BTRE-group (7/18, 39%) and 3 in the PROPHYLAXIS-group (3/10, 30%). Tumor dimension was >5 cm in 9 patients (9/18, 50%) and 3 patients (3/10, 30%) in the BTRE-group and PROPHYLAXIS-group, respectively. Ten patients (10/18, 55%) in the BTRE-group exhibited right-side localization of the brain lesion compared to 4 patients (4/10, 40%) in the PROPHYLAXIS-group.

All patients underwent brain surgery with total resection of the neoplastic lesion in 8 patients in the BTRE-group (8/18, 44%) and 5 patients in the PROPHYLAXIS-group (5/10, 50%). All patients were treated with the current standard oncologic treatment protocols, which included radiotherapy (RT) (age >70 years: 40 Gy; age <70 years: max 60 Gy) and chemotherapy (CT) (temozolomide for 6–12 cycles during and following RT) when indicated. If needed, anti-edema treatment with corticosteroids or mannitol was administered at the brain tumor diagnosis and discontinued after 1 week. Two patients (2/18, 11%) in the BTRE-group and five patients (5/10, 50%) in the PROPHYLAXIS-group experienced tumor relapse after RT/CT treatment. Tumor relapses were observed after an average time of 7 months (IQR: 6.5–8.5). In the BTRE-group, all patients with tumor relapses underwent second surgery followed by retrial of CT with temozolomide. In the PROPHYLAXIS-group, 3 patients underwent second surgery followed by CT with temozolomide, whereas 2 patients only underwent CT retrial with temozolomide. Brain tumor characteristics and treatments are summarized in Table 1.

### Neuropsychiatric Assessment

**Figure 1:**
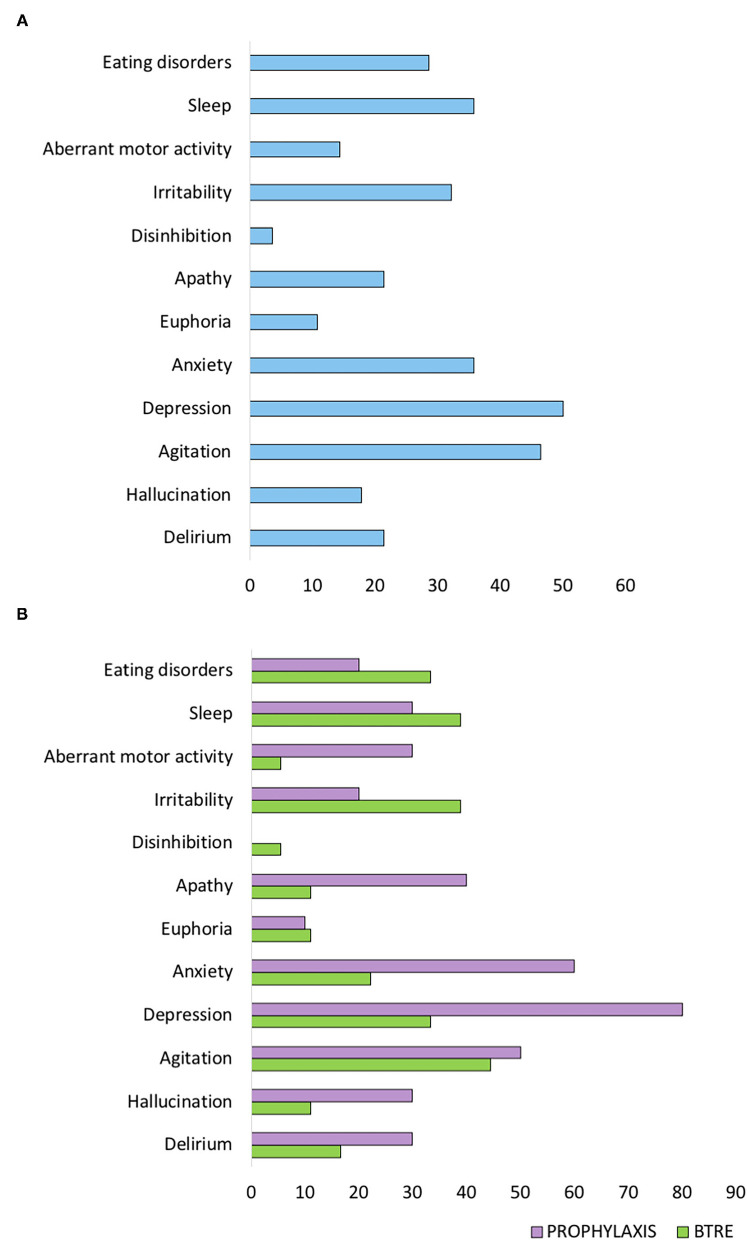
Neuropsychiatric Inventory (NPI-Q) scale score at 12 months follow-up visit. **(A)** NPI-Q sub-items scores of the entire study population. **(B)** NPI-Q sub-items scores of the two subgroups.

**Figure 2:**
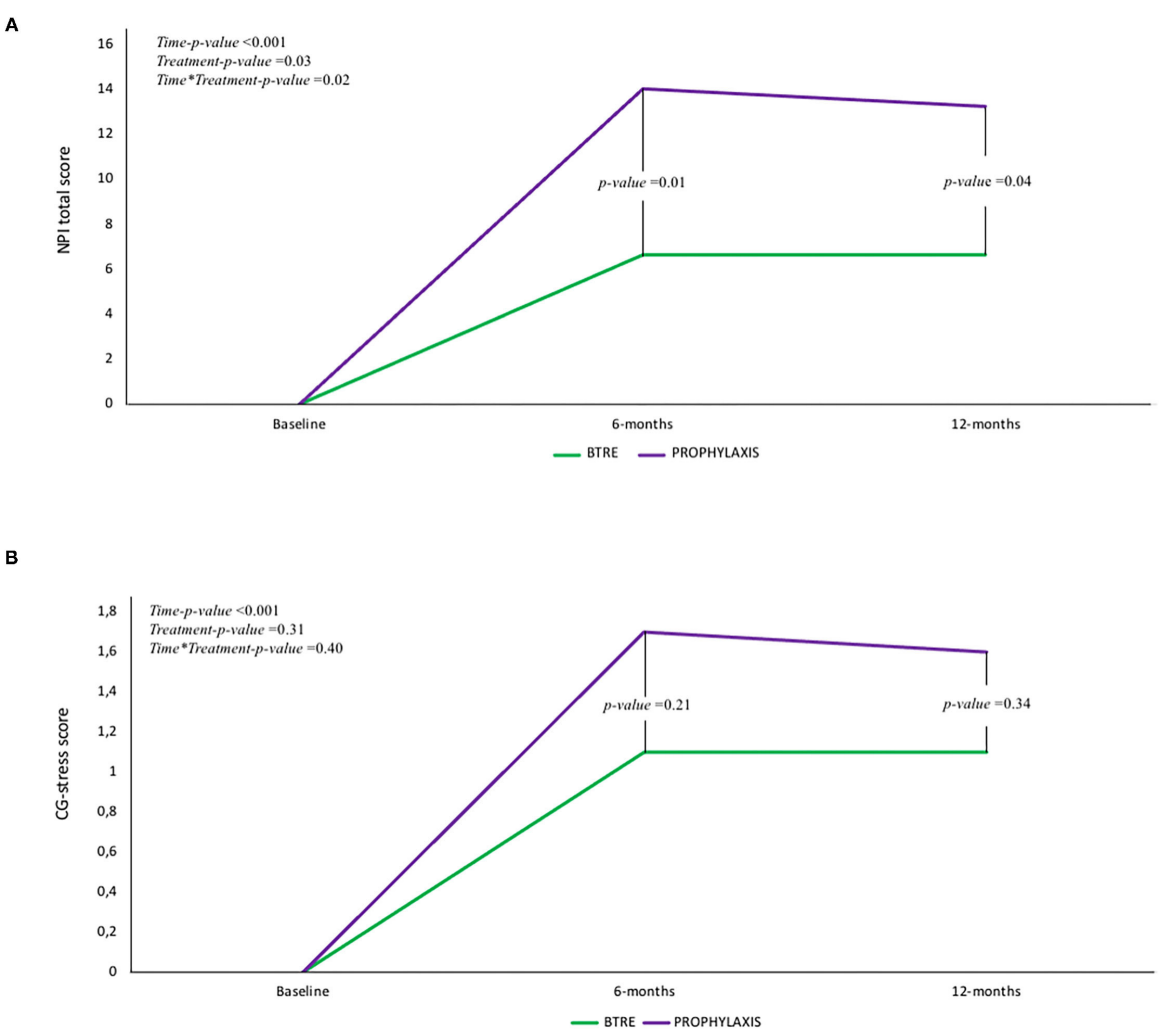
**(A)** Change in Neuropsychiatric Inventory (NPI-Q) total score in the two subgroups according to time. A multiplicative effect for the interaction between group treatment for time (*p* = 0.02) was observed. **(B)** Change in caregiver distress score (CDS) in the two subgroups according to time. For the CDS only a time-effect was observed whereas no additive or multiplicative effect was found.

At baseline, according to the enrollment criteria, both groups did not present neuropsychiatric symptoms as assessed by NPI-Q. The overall incidence of neuropsychiatric symptoms at 12 months follow-up visit is reported in Figure 1A. Agitation (46.4%), depression (53.6%), and anxiety (35.7%) were the most frequent neuropsychiatric signs associated with LEV treatment. However, compared to the BTRE-group, the incidence and the severity of neuropsychiatric symptoms in the PROPHYLAXIS-group were higher (see Figure 1B). Figure 2A shows changes in NPI-Q total scores in the two subgroups during follow-up. Interestingly, a multiplicative effect for the interaction between group treatment for time (*p* = 0.02) was observed. When plotting death and tumor characteristics as a covariate in the Mixed Model, the results were substantially unchanged (tumor characteristics *p* = 0.60; and death rate *p* = 0.79; AIC 486 vs. 479 in the full adjusted model). The use of symptomatic treatment with diazepam in case of agitation or anxiety was reported in 4 patients in the PROPHYLAXIS-group (4/10, 40%) and 1 patient in the BTRE-group (1/18, 6%) (median: 8 mg, IQR: 5–10).

Figure 2B shows CDS changes in the two subgroups according to time. For the CDS, only a time-effect was observed, whereas no additive or multiplicative effect was found. NPI-Q-scale and CG-stress scores were not different according to brain tumor characteristics.

### Mortality Risk

A total of seven deaths (7/28, 25%) were recorded in the entire study population, two of them (2/18, 11%) in the BTRE-group and five (5/10, 50%) in the PROPHYLAXIS-group (two-tailed Fisher's exact test *p* = 0.03). To assess the risk of mortality related to ASM, a Logistic Regression model was applied. In the PROPHYLAXIS-group, death could be associated with ASM with an OR = 8.00 (95% CI: 1.17–54.71). However, when in the Logistic Regression model, age and sex were considered, the association was no more statistically significant (PROPHYLAXIS-group OR = 6.10; 95% CI: 0.63–59.20). No significant association was found between tumor characteristics (i.e., tumor relapse, dimension, histology, surgical, and therapeutical approaches) and death (data not shown).

## Discussion

Our study indicates that patients treated with LEV as ASM prophylactic therapy show worse NPI-Q total scores and depression sub-scores when compared to patients treated with LEV in the context of BTRE. The mechanisms through which LEV can produce psychotic symptoms are largely unknown and not necessarily related to synaptic vesicle protein SV2A blockade. Supporting this notion, the recently introduced ASM Brivaracetam (BRV), a derivative of LEV/piracetam with a higher affinity to SV2A, has been associated with a lower incidence of psychiatric SEs than LEV. Moreover, LEV exhibits broad pharmacological effects due to the interaction with various receptors, such as α-amino-3-hydroxy-5-methyl-4-isoxazolepropionic acid (AMPA) glutamate receptors (AMPArs) (12). According to literature, AMPAr Inhibition by LEV is rapid and readily reversible (13). In addition, LEV modulated the presynaptic P/Q-type voltage-dependent calcium channel to reduce glutamate release (13).

α-amino-3-hydroxy-5-methyl-4-isoxazolepropionic acid glutamate receptors are highly expressed in glioblastoma and play a pivotal role in mediating the glutamate-related effects in gliomas. Experimental models show that high-grade gliomas release excitotoxic concentrations of glutamate, which has been shown to enhance tumor proliferation and migration (14). Stimulated AMPAr generates the cytoskeletal reorganization of glioma cells and has been shown to improve the detachment of cells from the extracellular matrix and glioma invasion (15).

Recent evidence confirmed that glutamate, the main excitatory neurotransmitter in the central nervous system, is a critical driver for tumor-associated seizures (16). Recent studies comparing patients with or without tumor-associated seizures demonstrated increased glutamate concentrations in tumors and peritumoral glioma tissues of seizure patients (17). In addition, increased expression of several glutamate receptor subtypes, such as AMPAr, has been shown in the reactive astrocytes of perilesional zones (18). Glutamate can also modulate the onset of psychiatric symptoms. Behavioral changes, agitation, anxiety, psychosis, aggressive behavior, and depression have been described in experimental and clinical settings assessing the use of AMPAr blockers (19). The higher expression of glutamate and increased level of AMPAr in BTRE patients may explain the reduced burden of LEV-related psychiatric SEs in the group. In particular, in BTRE patients, LEV blocks glutamate release and modulates AMPAr activation, thereby helping to halt the neuronal hyperactivation of the epileptic focus.

On the contrary, patients treated with prophylactic therapy with LEV do not exhibit increased glutamate expression or altered neuronal activation. In this context, the blockade of AMPAr may disrupt the brain homeostasis of glutamatergic transmission, thereby leading to the onset of psychiatric symptoms.

The LEV-related mechanism of action is not fully understood. The hypothesis of glutamate-dependent induction of network hyperactivity and the subsequent production of psychiatric symptoms in LEV-treated patients is a possibility. However, neuronal hyperactivity can also be independent of AMPAr overactivation.

According to a recent meta-analysis, ASM prophylaxis does not reduce the incidence of postoperative seizure in seizure-naïve brain tumor patients. On the contrary, ASM prophylaxis is associated with a relatively high rate of dermatological (rash), neurological (ataxia, decreased level of consciousness and aphasia), psychiatric (depression), and hematological (thrombocytopenia, electrolyte imbalance) SEs (up to 17–34%) (20). Supporting this evidence, we have shown that psychiatric SEs of prophylactic therapy, which are generally underestimated, may occur in patients with a brain tumor. In our cohort, the choice of prophylaxis treatment for BTRE was based on decisions of the clinicians shaped by divergent management policies for oncological patients. In particular, most of the PROPHYLAXIS-group patients were evaluated by clinicians with little experience in neuro-oncology and BTRE management. Undoubtedly, brain tumor features should be taken into account to assess the role of LEV in modulating the onset of psychiatric symptoms. Some studies stressed that lesion localization plays a role in psychiatric symptoms onset (21). In particular, neoplastic lesions in the frontal and parietal cortices and paralimbic structures have been generally associated with the occurrence of neuropsychiatric symptoms. In addition, some authors have suggested that the right localization of the lesion is associated with an increased rate of psychiatric symptoms (22). However, in our cohort, lesion sites and lesion lateralization were not different when comparing the two groups for NPI-Q total scores and depression sub-scores. No correlations with either tumor localization or tumor size were found, thereby supporting the assumption of a possible effect of LEV in the onset of psychiatric symptoms unrelated to brain tumor characteristics.

Neuropsychiatric symptoms can negatively affect social environment, such as family members and close friends, and performances of the patients in the activity of daily living. In addition, there are indications that brain tumor patients are at an increased risk for death by suicide even though this risk is lower if compared to patients with other oncological issues (23). Changes in personality and behavior, mood issues, hallucinations, and psychosis are challenging to be recognized in patients with brain tumors and have not been widely explored in literature (24). The use of specific scales and evaluation tools may help clinicians to generate more accurate psychiatric comorbidity evaluations. In this regard, the NPI-Q scale is a valid caregiver-rated measure of psychopathology in people with epilepsy.

The lack of evidence concerning the efficacy and tolerability of antidepressants (25) and antipsychotic treatments, the possible drug-to-drug interaction with CT (26), and the unknown benefits of cognitive-behavioral treatment (CBT) make the management of psychiatric symptoms in patients with brain tumors challenging. In this context, therapies associated with increased risk for suicide or suicide attempt, depression, or panic disorder should be avoided.

## Limitations

The study has several limitations. First of all, given the retrospective observational design of the study, the sample size of the PROPHYLAXIS-group is smaller than the BTRE-group. Hence, caution should be used when interpreting the data as they may not entirely represent brain tumor patients. Studies with larger cohorts are needed. A recent randomized control study in the United Kingdom (27) is set to address this issue and will provide information on the topic. However, our study provides clinicians with data to discuss the risk/benefit ratio of LEV prophylaxis with brain tumor patients.

## Conclusion

Several studies have demonstrated the efficacy and safety of LEV when used in the context of BTRE and as anti-seizure prophylactic therapy in brain tumor pathology. However, little data are available regarding the neuropsychiatric SEs of LEV intake in patients suffering from brain tumors. Our results support the importance of accurate epileptological evaluations in patients with brain tumors to identify and select who is most likely benefiting more from anti-seizure therapy.

## Data Availability Statement

The raw data supporting the conclusions of this article will be made available by the authors, without undue reservation.

## Ethics Statement

The studies involving human participants were reviewed and approved by G. d'Annunzio University of Chieti-Pescara, Protocol code 2098. 11/06/2020. Protocol Neurodem 26/7/2018, Emend 2/8/2018. The patients/participants provided their written informed consent to participate in this study.

## Author Contributions

FD: contributed to the conception and design of the study. AR and GE: organized the database. AD: performed the statistical analysis. FD, MO, and SS: wrote the manuscript and supervised all the data. All authors contributed to manuscript revisions, read, and approved the submitted version.

## Funding

This work has been supported by non-profit agencies [Italian Department of Health (RF-2013–02358785 and NET-2011-02346784-1), the AIRAlzh Onlus (ANCC-COOP), European Union's Horizon 2020 research and innovation program under the Marie Skłodowska-Curie grant agreement iMIND – no. 84166, the Alzheimer's Association – Part the Cloud: Translational Research Funding for Alzheimer's Disease (18PTC-19-602325), and the Alzheimer's Association – GAAIN Exploration to Evaluate Novel Alzheimer's Queries (GEENA-Q-19-596282)].

## Conflict of Interest

The authors declare that the research was conducted in the absence of any commercial or financial relationships that could be construed as a potential conflict of interest.

## Publisher's Note

All claims expressed in this article are solely those of the authors and do not necessarily represent those of their affiliated organizations, or those of the publisher, the editors and the reviewers. Any product that may be evaluated in this article, or claim that may be made by its manufacturer, is not guaranteed or endorsed by the publisher.
